# Expression of CD13 and CD26 on extracellular vesicles in canine seminal plasma: preliminary results

**DOI:** 10.1007/s11259-023-10202-1

**Published:** 2023-09-14

**Authors:** Alessandro Troisi, Magdalena Schrank, Ilaria Bellezza, Francesca Fallarino, Sara Pastore, John P. Verstegen, Camillo Pieramati, Alessandro Di Michele, Vincenzo Nicola Talesa, Marcelo Martìnez Barbitta, Riccardo Orlandi, Angela Polisca

**Affiliations:** 1 School of Bioscience and Veterinary Medicine, Università Di Camerino, Via Circonvallazione 93/95, 62024 Matelica (Macerata), Italy; 2 Department of Animal Medicine, Production and Health Università Degli Studi Di Padova, Agripolis Viale Dell‘Università - 35020 Legnaro, Padua, Italy; 3 Department of Medicine and Surgery, Università Di Perugia, P.Le Gambuli, 06132 Perugia, Italy; 4 Department of Veterinary Medicine, Università Di Perugia, Via San Costanzo 4, 06126 Perugia, Italy; 5 TherioExpert LLc. and College of Veterinary Medicine, University of Nottingham, Nottingham, UK; 6 Department of Physics and Geology, University of Perugia, Via Pascoli, 06123 Perugia, Italy; 7 Tyrus Veterinary Clinic, Via Aldo Bartocci, 1G, 05100 Terni, Italy; 8 Integral Veterinary Reproductive Service URUGUAY (SRVI_UY); Postgraduate Program, Faculty of Veterinary Medicine - University of Republic (UdelaR - UY), Faculty of Veterinary Medicine - University of Republic (UdelaR - UY), Uruguay, Uruguay

**Keywords:** Canine semen, Extracellular vesicles, Dipeptidyl peptidase IV, Aminopeptidase-N

## Abstract

Canine seminal plasma is a complex fluid containing proteins, peptides, enzymes, hormones as well as extracellular vesicles that are involved in many physiological and pathological processes including reproduction. We examined the expression of the extracellular vesicles surface antigens Aminopeptidase-N (CD13) and Dipeptidyl peptidase IV (CD26) by flow cytometry. For this study, third fraction of the ejaculate, from fertile adult male German Shepherd dogs, was manually collected twice, two days apart. FACS analyses revealed that CD13 and CD26 are co-expressed on the 69.3 ± 3.7% of extracellular vesicles and only a 2.0 ± 0.5% of extracellular vesicles express CD26 alone. On the other hand, 28.6 ± 3.6% of seminal EVs express CD13 alone. Our results agree with the hypothesis that CD26 needs to be co-expressed with other signal-transducing molecules, while CD13, can perform functions independently of the presence or co-expression of CD26. The results obtained in normal fertile dogs could represent physiological expression of these enzymes. Therefore, it would be interesting to carry out further studies to evaluate the expression of CD13 and CD26 on extracellular vesicles as biomarker for prostate pathological condition in dogs.

**Supplementary Information:**

The online version contains supplementary material available at 10.1007/s11259-023-10202-1.

## Introduction

The seminal plasma can be considered not only as a passive carrier for spermatozoa but also a rich source of bioactive and immunomodulatory factors involved in some reproduction processes. Mammalian semen is a rich source of extracellular vesicles (EVs) (Höög and Lötvall 2015), membrane-enclosed particles secreted into the extracellular space or fluids by all mammalian cell (Théry et al. 2002). EVs represent important vehicles of intercellular communication and an essential mechanism for development and maintenance of multicellular organisms (Turturici et al. 2014). The Executive Committee of the International Society of Extracellular Vesicles defined EVs as particles ranging from 30 to 2000 nm in diameter (Théry et al. 2002; Llobat 2021; Théry et al. 2018). The prostate gland secretes EVs that, based on their tissue specificity, were historically called prostasomes (Ronquist and Brody 1985). However, due to the increasing interest of the scientific community on EVs research the MISEV guidelines, selected the term “extracellular vesicle” (EV) as the generic term for particles naturally released from the cell that are delimited by a lipid bilayer and cannot replicate, independently of their tissue of origin. Thus, we choose to indicate prostasomes as seminal EVs in the current paper (Théry et al. 2018). Since the first observation of EVs intervention in intercellular communication, many studies have reported the influence of these vesicles in female and male reproduction. In fact, the semen of several mammals, such as man (Brody et al. 1983), horse (Minelli et al. 1998), bull (Frenette et al. 2002), boar (Piehl et al. 2006), rabbit (Castellini et al. 2012), dog (Zelli et al. 2013; Goericke-Pesch et al. 2015) and cat (Polisca et al. 2015) contain vesicles of different composition and origin. Moreover, seminal EVs are involved in the process of mammalian fertilization in several ways; they can regulate many sperm cell functions including sperm motility (Agrawal and Vanha-Perttula 1987; Han et. al 2020), acrosome reaction (Siciliano et al. 2008), sperm cell capacitation (Aalberts et al. 2013), and possess antibacterial and antioxidant properties (Carlsson et al. 2000; Saez et al. 1998). Another main function of seminal EVs is to protect sperm cells from the female immune system during their transport to the ovum (Tarazona et al. 2011). Finally, seminal EVs have been proposed as a novel biomarker for human infertility and prostate cancer in human (Vickram et al. 2020; Park et al. 2016).

EVs are surrounded by a membrane with an unusual lipid composition and a specific cholesterol/phospholipidic ratio (Bellezza et al. 2005). Studies have reported about 3500 protein species isolated from EVs secreted by different cell sources (Haraszti et al. 2016) and functional mRNA from approximately 1300 genes (Valadi et al. 2007).

Proteomic analyses revealed that EVs from different mammalians show characteristic protein composition which is related to peculiar physiological functions (Ronquist 2015; Baskaran et al. 2020; Poliakov et al. 2009; Ronquist et al. 2013; Leahy et al. 2020; Gaitskell-Phillips et al. 2022; Wang et al. 2022).

Among EVs proteins, dipeptidyl peptidase IV (CD26) is a transmembrane serine protease located on the surface of the plasma membrane of a variety of cells as well as of EVs (Carlsson et al. 2006). CD26 is an exopeptidase that removes N-terminal dipeptides from polypeptides with unsubstituted N-termini. It acts on protein with proline, hydroxyproline, or alanine as penultimate residue: the greatest hydrolysis rate is with proline, when the third residue is neither proline nor hydroxyproline (Vanhoof et al. 1992). CD26 is pivotal for immune functions as a T-cell activation molecule and a regulator which may aid in the protection of the spermatozoa in the genital tract (Schrimpf et al. 1999; Hu et al. 2022). Moreover, CD26 is transferred to sperm from prostate derived EVs (formerly prostasomes) through a pH-dependent fusion mechanism, the phenomenon is more evident at pH 5 than at higher pH values (Arienti et al. 1997). These peptidases have also been proposed as contributors to an additional mechanism of cell-to-cell interaction and as signalling molecules (Carlsson et al 2006). Other roles proposed are in the metabolism of cytokines and growth factors (Wilson et al. 1998 and Vanhoof et al. 1995).

Aminopeptidase-N (CD13) is a surface antigen found in several cell types and, like CD26, it has also a peptidase activity. It is a Zn^2+^ dependent membrane-bound ectopeptidase that preferentially degrades proteins and peptides with a N-terminal neutral amino acid (Wickström et al. 2011). Mammalian CD13 is an important player in many physiological processes such as sperm motility, cell adhesion and immune cells chemotaxis (Ronquist 2015). In particular, CD13, which has been regarded as enkephaline-degrading enzyme, has been involved in sperm motility, mainly by the regulation of endogenous opioid peptides. Although clinical studies have suggested that several diseases, such as infertility, may depend on alterations in the expression/activity of these enzymes (Fernandez et al. 2002; Bosler et al. 2014), their role in regulating male fertility remains poorly understood. It has been proposed that these membrane-bound peptidases may serve as negative loops in controlling concentration of bioactive peptide-signaling pathways in human semen (Carllson et al. 2005).

Both sperm motility and protection from female immunoreaction during sperm transport in the genital tract appear to be essential for reproduction and are currently the major reliable factors of potential fertility. As previously illustrated, these functions in humans are also attributed, to CD13 and CD26 expressed on the surface of EVs.

There are anatomical and physiological similarities between the human and canine prostate. In particular, the canine and human prostate have a similar ovoid, bilobed structure and are both situated at the base of the bladder, encompassing the proximal urethra. In addition, as far as aspects of pathology are concerned, the dog is the only animal species that, with advancing age, develops benign prostatic hyperplasia (BPH) (Ryman-Tubb et al. 2022).

Since some similarities exist between the canine and human prostate, we aimed at analyzing whether the same correlations might also exist in seminal EVs (Romagnoli et al 2022).

As previously described, the expression of CD13 and CD26 haave been found in EVs from humans and many animal spiecies and for this reason, the aim of this study was to evaluate CD13 and CD26 expression in canine prostate-derived EVs by flow cytometric technique to define potential reference values of CD13 and CD26 expression in healthy dogs. To our knowledge, this is the first attempt to assess these expressions in dog semen.

## Materials and method

### Reagents

Fluorescein isothiocyanate (FITC)‐conjugated mouse monoclonal antibody anti CD13(MHCD1320) and Phycoerythrin (PE)- conjugated mouse monoclonal antibody anti CD26 (CD2604) were obtained by Life Technology (Carlsbad, CA, USA).

### Animal samples

The study was approved by the Ethical Committee of the University of Perugia (Prot.n.125706/ June 2021) and performed, with owner consent, in accordance with Italian laws and EU directives as a part of normal veterinary clinical practice. The animals used in this study (*n* = 25) were fertile adult male German Shepherd dogs and confirmed healthy based on history and clinical examination which included blood analysis (complete blood count analyses and biochemical analyses), andrological evaluation, and ultrasound exam of the prostate and testis (MyLab 30 Gold system Esaote; Genoa, Italy equipped with a 5.5–7.5 MHz microconvex probe).

Semen was collected as previously described (Johnston 1991), from 25 German Shepherd dogs, aged from 2 to 4.5 years. In particular, for antibody specificity and reactivity samples from 9 out of 25 dogs were analysed; for the obtainment of ultracentrifuged EVs samples from 7 out of 25 dogs were used; to evaluate the repeatability of the trait, semen was collected twice from the same dog two days apart, from 9 out of 25 dogs. The detection of CD13 and CD26 expression has been performed in each individual sample.

Eight milliliters of blood were used to isolate monocytes and platelets using the following procedures. Peripheral blood mononuclear cells (PBMC) were isolated from leucocyte-enriched (buffy coats) canine peripheral blood. Blood samples were diluted with RPMI 1640 (Gibco, Milan, Italy) without serum, layered over Histopaque 1077 (Sigma-Aldich, st. Luis, Missouri, USA), and centrifuged at 400 g for 20 min in an Eppendorf centrifuge (Milan, Italy). The layer containing the mononuclear cells at the interface between plasma and Histopaque was recovered and washed twice with RPMI 1640. Viable PBMCs were counted by the trypan-blue exclusion technique and the density was adjusted to 1 × 10^6^ cells/ml with RPMI 1640. Cells were then used for the experiments.

To obtain a platelet rich plasma, blood was centrifuged at 500 × g for 10 min. The supernatant was removed for further centrifugation at 3500 × g for 7 min, without brake. The supernatant was removed, and the platelet rich pellet was resuspended in PBS and used for the analyses.

The first, second and third fractions of the ejaculate were separated into three vials. Immediately after collection, each third fraction was centrifuged at 800 × g for 10 min to eliminate spermatozoa and supernatant was frozen at -80 °C until use, and each second fraction was subjected to macro and microscopic examination. Total and progressive motility were assessed subjectively through the observation of 10 µl of fresh semen placed on a glass slide (37.5 °C) under a 100 × phase-contrast microscope (Nikon®-Y2B-Ph-11 Tokyo, Japan).

Sperm concentration was determined using a Burker counting chamber (Merck, Leuven, Belgium) after a 1:100 dilution with water. The percentage of live and dead spermatozoa and the spermatozoa morphology were examined on eosin–nigrosin-stained smears. At least 100 spermatozoa were evaluated per slide.

### EVs isolation

EVs isolation was performed according to Mezzasoma et al. 2022 with slight modifications. Briefly, the centrifuged third fraction was thawed, diluted (1:1 v/v) with 30 mM Tris–HCl and 130 mM NaCl buffer pH 7.6 centrifuged at 5000 g for 30 min at 4 °C to eliminate cell debris and residual spermatozoa. The pellet was discarded, and the supernatant was centrifuged at 100,000 × g at 4 °C in an Optima TLX ultracentrifuge with a swinging-bucket 60Ti rotor (Beckman Coulter, Brea, CA, USA) for 2 h at 4 °C. The pellet containing EVs was suspended in PBS and diluted to obtain a final concentration of 1.0 – 1.5 mg of protein per ml. The EVs preparation was stored at -80 °C until use. Protein concentration was determined using Bradford assay (Bio-Rad-Lab, Hercules, CA, USA).

### Western blotting

Isolated EVs were resuspended in Laemli sample buffer and sonicated tree times (3 × 10 s, 30 s pause) on ice. The obtained whole protein extracts were separated on a 12% sodium dodecyl sulfate–polyacrylamide gel electrophoresis (SDS-PAGE) and transferred on nitrocellulose membrane. Non-specific binding sites were blocked in 5% non-fat milk for 1 h at room temperature. The membranes were blotted overnight at 4 °C with anti-Alix antibody (1:500) (#E-AB-62330, Elabscience, Houston, TX, USA), anti-CD81antibody (1:500) (#E-AB-63817, Elabscience, Houston, TX, USA), anti-TSG101 antibody (1:200) (#sc-7964, Santa Cruz Biotechnology, Dallas, TX, USA) and anti-calnexin antibody (1:500) (#E-AB-30723, Elabscience, Houston, TX, USA) all diluted in 5% non-fat milk. After washing with TBST, blots were incubated for 1 h at room temperature with the appropriated HRP-conjugated secondary Abs (1:5000) and chemiluminescence was detected through an iBright™ CL1500 Imaging System (Thermo Fisher Scientific, Waltham, MA, USA). The antibodies used for EVs characterization were raised against a human epitope, thus an amino acid sequence alignment of human and canine Alix, Calnexin, TSG101 and CD81 was performed using the EMBOSS Matcher software (https://www.ebi.ac.uk/Tools/psa/emboss_matcher/). These analyses revealed an identity of 89.1% for Alix, 94.9% for Calnexin, 97.7% for TSG101, and 97.0% for CD81, strongly suggestive of an interspecies cross-reactivity. EVs isolated from human amniotic fluid-derived stem cells (Mezzasoma et al. 2022) and human monocytic THP-1 whole protein extracts (Mezzasoma et al. 2022) were used as controls.

### Scanning Electron Microscopy (SEM) analysis

EVs were fixed in 4% paraphormaldehyde for 30 min at room temperature, washed with water, sedimented on glass coverslips and then allowed to dry at room temperature. SEM images were obtained using a field emission gun electron scanning microscope (LEO 1525 Zeiss; Thomwood NY, USA) with Cr metallization using a high-resolution sputter 150 T ES-Quorum apparatus (24 s, sputter at a current of 240 mA). Chromium thickness was ~ 22 nm.

### Flow cytometric analysis (FACS)

Isolated EVs (300 μl), or third fractions (300 μl), were stained with anti-CD13 (FITC) or anti-CD26 (PE) antibody for 1 h at room temperature, then diluted 1:2 with PBS before FACS analyses. At least 20,000 EVs were analysed per sample using a LSR Fortessa (BD Biosciences, CA, USA) flow cytometer. The flow cytometer was equipped with solid laser with a 488-nm filter a 95% reduction filter, a 530/505 nm band pass filter and a 575/550 nm band pass filter to detect CD13 (FITC) and CD26(PE) stained EVs, respectively. Data were collected on log scale histograms at a rate of 5,000–10,000 events per second. Populations were gated using forward/side scatter (linear scale) to identify EVs and then by their fluorescent properties (log scale) in accordance with fluorophore’s properties. Control samples of each stain, single and in combination were analysed prior to experiments to establish the gates for each stain and then prior to analyses. Calibration of the flow cytometer was performed using CST beads (BD bioscience) consisting of equal quantities of 3-µm bright, 3-µm mid, and 2-µm dim polystyrene beads. Each EVs was represented by a point in a rectangular co-ordinate system, based on its light-scattering properties (Mezzasoma et al. 2022). The position and dimension of the gate were placed in line with that of purified EVs. A detailed MIFlowCyt-EV framework has been included as supplementary file (Supplementary file 1).

#### CD13 and CD26 antibody validation

**Fig. 1:**
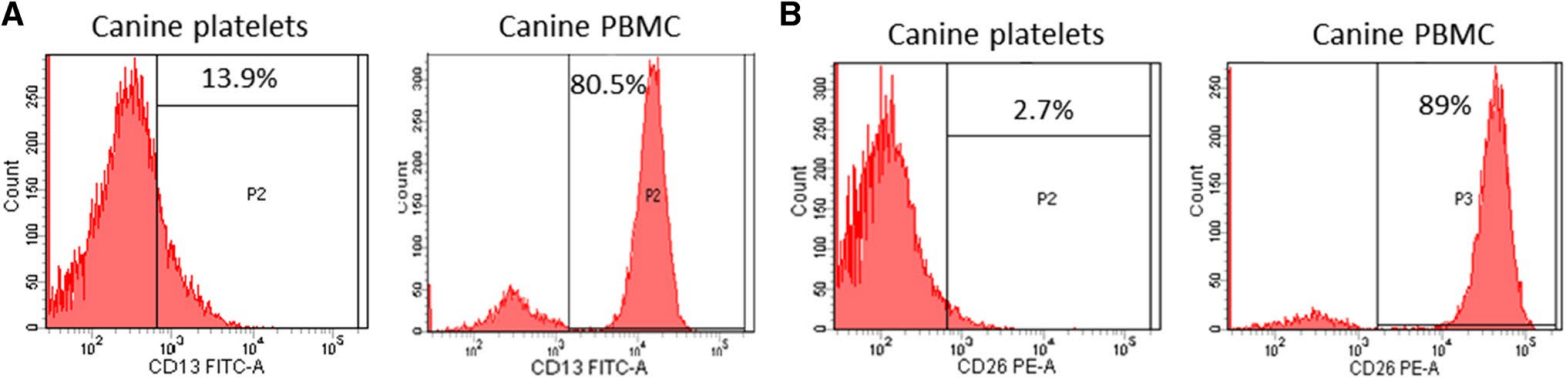
Canine platelets and PBMC were isolated and stained with FITC-labelled anti-CD-13 antibody (**A**) or PE-labelled anti CD-26 antibody (**B**). Fluorescent signals were analyzed by FACS. Numbers in the histograms represent the percentage of events measured in the gated area

CD13 and CD26 antibody are raised against a human epitope, thus an amino acid sequence alignment of human and canine CD13 and CD26 was performed using the EMBOSS Matcher software (https://www.ebi.ac.uk/Tools/psa/emboss_matcher/). These analyses revealed an identity of 77.7% and 88.7% and a similarity of 87.9% and 94.4%, respectively, strongly suggestive of an interspecies cross-reactivity. The specificity of anti-CD13 and anti-CD26 antibody were validated using canine PBMCs and platelets as positive and negative controls, respectively. FACS analyses showed that CD13 (Fig. 1A) and CD26 (Fig. 1B) antibodies used in the study, were capable of binding canine PBMC but not canine platelets, as expected. Protein similarity and validation of antibody targets confirmed the specificity of the anti-CD13 and anti-CD26 antibodies in recognizing canine proteins.

### Statistical analyses

FACS results were inserted into a spreadsheet (Microsoft Excel) as percentage of population expressing both CD13 and CD26, only CD13, only CD26, or expressing neither one. Statistical analysis was performed by “R” software (R Core team 2021).

Data repeatability was assessed by a linear model (“lme4” library: Linear Mixed-Effects Models using 'Eigen' and S4) fitting dog as a random effect: repeatability was estimated as the ratio *dog variance / (dog variance* + *error variance)*. The effect of age was estimated by the same “lme4” library, setting up a mixed linear model: the fixed effect was age as a covariate and the random effect was dog. The correlations between the expression of CD13 and CD26 was assessed by “rmcorr” (Repeated Measures Correlation) library of “R” (Bland and Altman 1995).

## Results

### Canine third fraction contains EVs

To characterize EVs from dog semen, we analysed German Sheppard dogs in good reproductive health condition. In particular, total ejaculate volumes, total number of spermatozoa, total sperm motility, sperm morphology and viability were within the range published for this specie for each dog (Johnston 1991). The mean with standard deviation values and the reproductive history (pregnacy rate and litter size) has been reported in Table 1 and in Table 2.

**Table 1:** Values of seminal characteristics in twenty-five dogs. Data are reported as means ± SD

	Media	± SD	Range(Johnston1991)
Total ejaculate volumes ml	11,2	1,8	1 to 30
Total number of spermatozoa × 10^6^	503,8	86,1	300 to 2000
Total sperm motility %	87,2	4,3	> 70%
Progressive sperm motility %	80,8	5,3	> 70%
Abnormal sperm morphology %	7,3	2,7	< 30%

**Table 2:** Reproductive history: litter size values are expressed as mean ± SD

Dog	Year	Previous litter	Litter size
A	4	3	7,33 ± 0,58
B	4,5	8	5,50 ± 2,56
C	4,5	7	5,60 ± 1,29
D	2,5	4	6,25 ± 1,50
E	2	5	5,40 ± 3,05
F	2	5	5,20 ± 1,48
G	4,5	4	5,50 ± 0,71
H	4	3	3,00 ± 2,00
I	4,5	6	7,40 ± 3,01

**Fig. 2:**
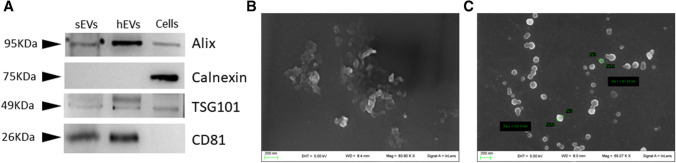
Canine third fractions were used to isolate and characterize EVs. (**A**) Western blotting analyses of the indicated EVs markers in canine seminal EVs (sEVs). EVs isolated from human amniotic fluid-derived stem cells (hEVs) and human monocytic THP-1 whole protein extracts (cells) were used as controls. SEM images of EVs in ultracentrifuged (**B**) and non-ultracentrifuged **C** canine third fraction

EVs isolated from canine third fraction were characterized by western blotting and SEM analyses (Fig. 2). Isolated canine seminal EVs (sEVs) express Alix, TSG101 and CD81, known EVs markers (Théry et al. 2018), while the expression of calnexin, an endoplasmic reticulum marker, was not detected (Fig. 2A). These results indicate the absence of co-isolated cellular debris. SEM analyses showed the presence of round-shaped vesicles with a diameter of approximately 100 nm (Fig. 2B and C). However, while ultracentrifuged samples contain agglomerated EVs with an apparently rough surface (Fig. 2B), the EVs from non-ultracentrifuged samples appear round shaped and well separated (Fig. 2C).

### EVs from canine third fraction express CD13 and CD26

**Fig. 3:**
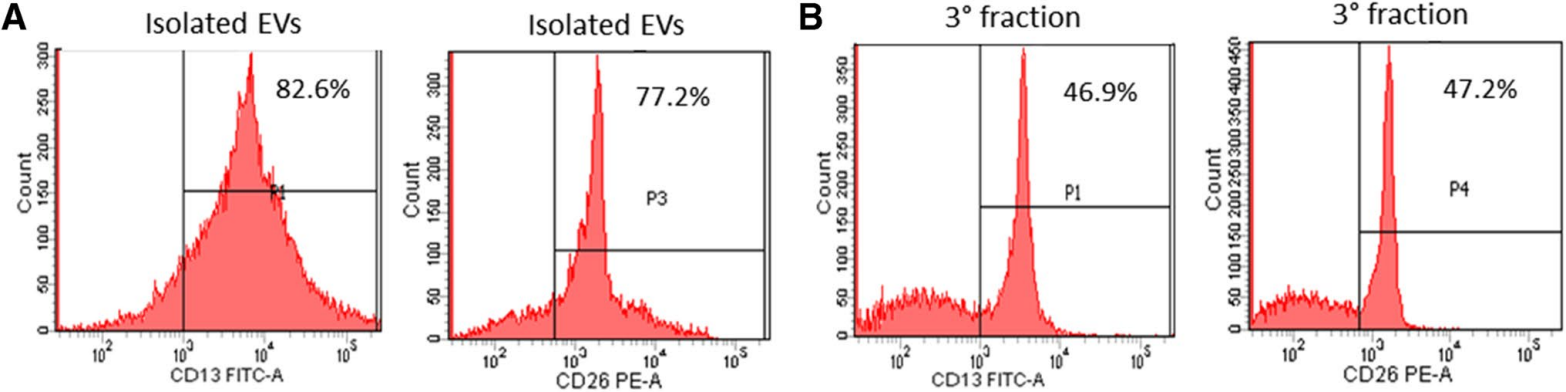
Canine third fractions were used to isolate EVs by sequential centrifugation steps (**A**) or left untreated (**B**) and stained with FITC-labelled anti-CD-13 antibody or PE-labelled anti CD-26 antibody. Fluorescent signals were analyzed by FACS. Numbers in the histograms represent the percentage of events measured in the gated area

The presence of CD13 and CD26 on seminal EVs surface was determined by FACS analyses. We found that seminal EVs isolated by sequential centrifugation steps expressed both CD13 and CD26 on their surface (Fig. 3A). CD13 and CD26 were also immunodetected on seminal EVs from the whole third fraction, which does not undergo the ultracentrifugation step (Fig. 3B).

The study of EVs is usually performed by their isolation through sequential ultracentrifugation steps which leads to a good ratio between specificity and recovery (Théry et al. 2018). Since it has been reported that ultracentrifugation can negatively affect EV integrity (Linares et al. 2015), and accordingly to our SEM analyses (Fig. 2B), we omitted the ultracentrifugation step prior to FACS analyses.

**Fig. 4:**
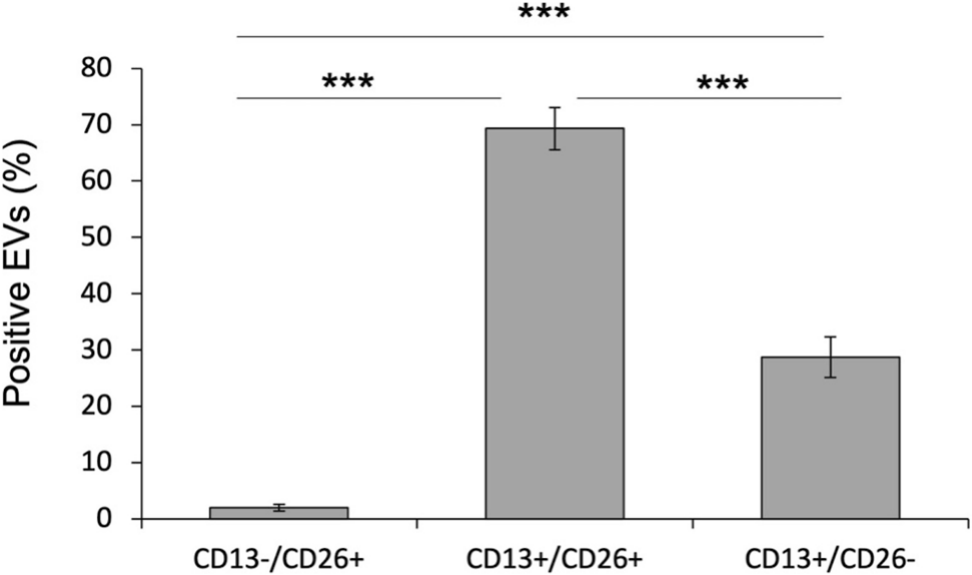
Canine third fractions, stained with FITC-labelled anti-CD-13 antibody and PE-labelled anti CD-26 antibody, were subjected to FACS analyses. Percentage of EVs labelled with CD13 only, CD26 only and both CD13 and CD26 was reported. (*n* = 18). *** *p* < 0.001

Simultaneous analysis of both antigens revealed that, the majority of the examined seminal EVs population concomitantly expressed CD13 and CD26 markers, whereas only a 2.0% expressed CD26 alone (*p* < 0.001)(Fig. 4).

**Fig. 5:**
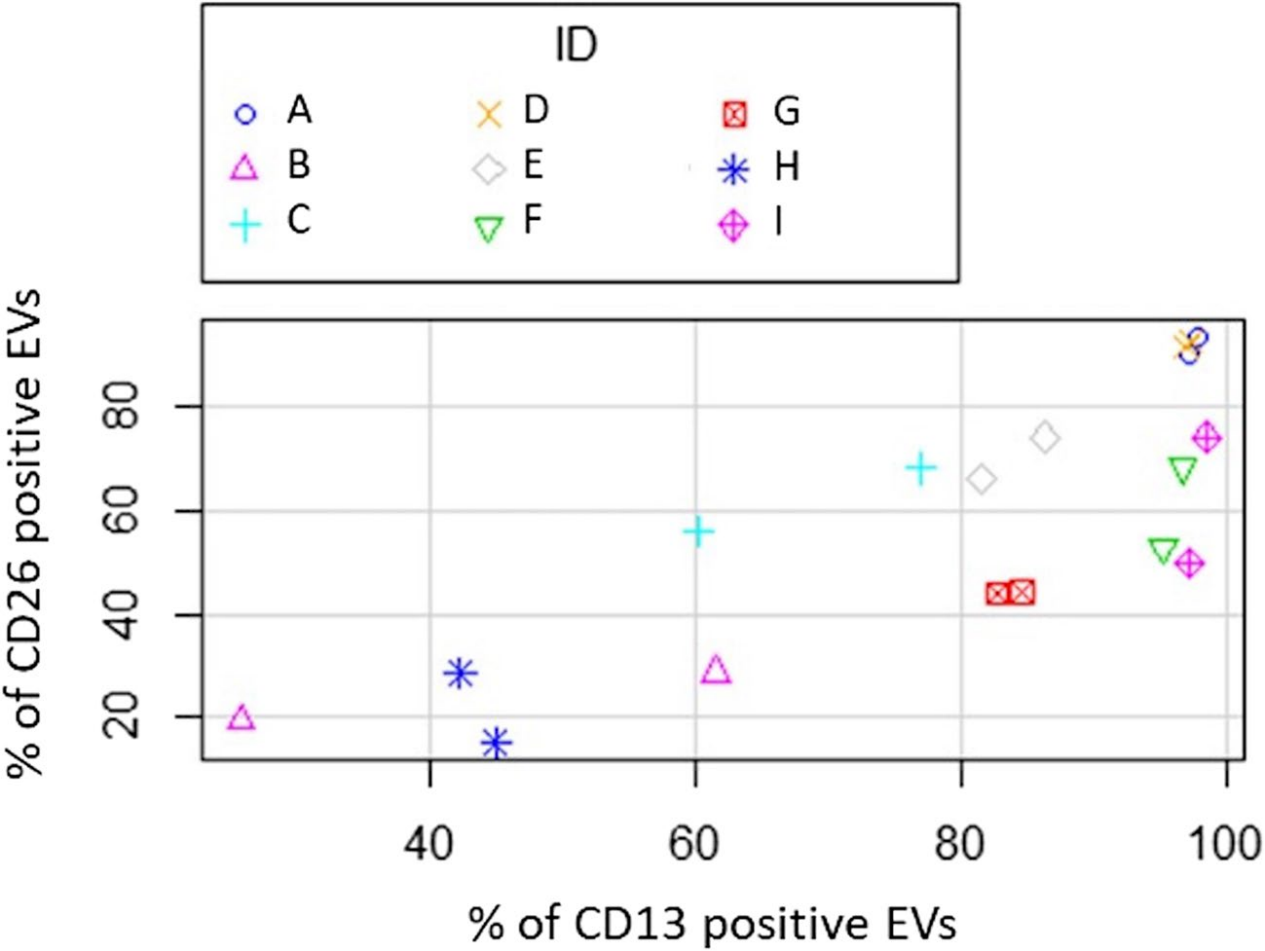
Percentage of populations expressing CD13 (horizontal axis) or CD26 (vertical axis) in the two samples of each dog is identified with a different uppercase letter

In order to assess data repeatability, we measured CD13 and CD26 expression in samples taken from the same dog two days apart. Repeatability estimates were 0.901 for the population double expressing CD13 and CD26, 0.604 for the population expressing only CD13, and 0.756 for that expressing only CD26. The good agreement between the two successive samplings can be endowed by the finding that 7 out of 9 animals gave very similar results (Fig. 5). A positive correlation between the expression of the two antigens was also clear: repeated measure correlation estimated a significant coefficient of 0.54 (confidence interval 95%: from 0.12 to 0.80; *P* < 0.05).

## Discussion

EVs, known to play a key role in intercellular communication, interact with spermatozoa by binding specific sperm membrane receptors, fusion, and content transferring (Roca et al. 2021). In fact, EVs, after a CD26-mediated fusion process (Minelli et al. 1998), may transfer their content, including of CD13, into spermatozoa (Arienti et al. 1997), thus taking part in viability and activation of the sperm cells especially as they enter the female genital tract. Our results have identified, for the first time, the expression of CD13 and CD26 on EVs from canine seminal plasma by means of cytofluorimetric analyses. Our method is based on the use of human specific antibodies; which reactivity toward canine antigen has been validated by staining blood samples drawn from the subjects of the experimentation. Moreover, the good agreement between the two successive samplings also indicate that the detection of CD13 and CD26 antigens is not affected by the timing or the frequency of sampling.

The presence of CD13 and CD26 markers in EVs is in accordance with what has been reported in humans (Carlsson et al. 2006; Arienti et al. 1997). In veterinary medicine, CD26 and CD13 expression has been analysed mostly by means of enzymatic activity (Castellini et al. 2012; Agrawal and Vanha-Perttula 1987; Minelli et al. 1999; Viudes-De-Castro et al. 2014; Alvarez-Rodriguez et al. 2019) which gives an average result of the whole sample. Notably, flow cytometric analysis, analysing the single particle, allowed us to evaluate the percentage of CD13 and CD26 positive EVs and the co-expression of the two protein on the same EVs.

Reinhold and colleagues (2006) reported the simultaneous presence of CD26 and CD13 on T cells and hypothesized that it may be advantageous to achieve full activity, in line with our observation that CD13 and CD26 are co-expressed in the vast majority of EVs isolated from dog semen. Furthermore, this co-expression could confirm the hypothesis that CD26 can be co-expressed with other signal-transducing molecules (Minelli et al. 1999) which in our experimental model would be represented by CD13.

Our results showed that the one third of EVs contain CD13 but not CD26, thus suggesting that CD13 can perform functions independent of the presence / co-expression of CD26. It has been indeed reported that CD13 possesses a moonlighting function which goes beyond its mere enzymatic activity (Mina-Osorio 2008), implying that it does not require a unique protein partner to achieve its functions. For example, CD13 signalling ability seems to depend on its association with auxiliary proteins that still needs to be identified (Mina-Osorio 2008).

CD13 and CD26 are known to be involved in immunomodulatory mechanisms (Reinhold et al. 2006), in sperm motility (Mina-Osorio 2008; Subirán et al. 2010), and in the fusion process that occurs between EVs and spermatozoa (Minelli et al. 1998; Arienti et al. 1997). It has been shown that the fusion between stallion EVs and spermatozoa requires the expression of CD26 on the surface of EVs. The fusion process leads to the enrichment of spermatozoa in CD26 expression and activity (Minelli et al. 1999), thereby providing sperm cells with enzymatic activities needed for a better accomplishment of the fertilization process. Therefore, it can be hypothesized that the presence of CD26 on canine EVs could play a role in fusion process also in dogs.

Furthermore, Khatun et al. (2018) reported that lowering CD13 activity in mouse epididimal spermatozoa slightly increased sperm motility and the percentage of high-speed spermatozoa while decreasing the percentage of slow speed spermatozoa. It is to underline that the observed increase in sperm motility adversely affected early embryonic development prompting the Authors to suggest the lowering of CD13 as contraception strategy (Khatun et al. 2018). Accordingly, exogenous CD13 supplementation increased the number of spermatozoa with sperm defects, suggesting that increased CD13 levels may disturb cellular homeostasis resulting in adverse effects (Khatun et al. 2017). These reports indicate that seminal CD13 levels should be strictly regulated to maintain physiological sperm functions.

Our results obtained in normal fertile dogs, represent the physiological expression of CD13 and CD26 enzymes on EVs surface, and could help in the decoding of EVs functional role in healthy dog seminal plasma.

In human medicine, a changes in CD13 and CD26 expression correlates with prostate disease (Bogenrieder et al. 1997; Biggs et al. 2016; Runsheng et al. 2008; Liu et al. 2004). In particular, CD26 was previously found to be strongly expressed on prostasomes (Vanhoof et al. 1992) as well as on the luminal surface of benign prostatic epithelial cells and on the surface of prostate cancer cells in nearly 100% of the cases (Dinjens et al. 1990). However, in the presence of metastasis CD26 expression was reduced or absent in 50% of cases (Dinjens et al. 1990) while CD 13 showed very low or no activity (Carlsson et al. 2003), suggesting that loss of these ectoenzyme may be involved in the development of metastatic disease (Dinjens et al. 1990; Carlsson et al. 2003). Finally, more recently, Agarwal et al. (2022) reported that the number of EVs increases in the prostatic cancer (PCa) cells in comparison with benign prostatic hyperplasia cells and, among the PCa cells, they bear a positive correlation with the Gleason score. These results indicate that EVs have the potential to become a biomarker.

In conclusion, we found that CD13 and CD26 can be immunodetected on EVs from the whole third fraction, which does not undergo the ultracentrifugation step, and that the one third of EVs contain CD13 but not CD26, thus suggesting that CD13 can perform functions independent of the presence / co-expression of CD26.

From a prostatic point of view men and dogs have several things in common. In both species the.

prostate is an essential accessory sex gland located on the bladder neck. Its secretions constitute the majority of the ejaculate volume and provide a significant contribution to sperm survival, maturation, transport and fertilization (Johnston et al. 2001). For this reason, it could be hypothesized that, also in dog, any modifications of the expression of CD13 and CD26 could be correlated with prostatic pathological conditions and represent a biomarker to be used as a diagnostic tool particularly for early and noninvasive diagnosis of BPH and prostatic neoplasia. Further studies are needed to evaluate EVs number and the expression of CD13 and CD26 on EVs surface in dogs suffering from benign prostatic hyperplasia or prostate cancer.

### Supplementary Information

Below is the link to the electronic supplementary material.

Supplementary file1 (DOCX 18 KB)

## Data Availability

The data that support the findings of this study are available from the corresponding author upon reasonable request.
