# Supplementary material for: Expression of CD13 and CD26 on extracellular vesicles in canine seminal plasma: preliminary results
Source: Vet Res Commun. 2023 Sep 14;48(1):357–66. doi: 10.1007/s11259-023-10202-1 (PMC10811140; doi:10.1007/s11259-023-10202-1)
Supplement: Supplementary file 1 — Supplementary file1 (DOCX 18 KB) [file 11259_2023_10202_MOESM1_ESM.docx]

**SUPPLEMENTARY INFORMATION (SI)**

**EXPRESSION OF CD13 AND CD26 ON EXTRACELLULAR VESICLES IN CANINE SEMINAL PLASMA: PRELIMINARY RESULTS.**

^a^ Troisi Alessandro; ^b^ Schrank Magdalena, ^c^ Bellezza Ilaria; ^c^ Fallarino Francesca; ^d^ Pastore Sara; ^e^ Verstegen John P.; ^d^ Pieramati Camillo; ^f^ Di Michele Alessandro; ^c^ Talesa Vincenzo Nicola, ^d^ Martinez Barbitta Marcelo, ᶢ Orlandi Riccardo; ^d^ Polisca Angela*.

^a^ School of Bioscience and Veterinary Medicine, Università di Camerino, Via Circonvallazione 93/95 62024 Matelica (Macerata), Italy

^b^ Department of Animal Medicine, Production and Health Università degli Studi di Padova, Agripolis Viale dell'Università - 35020 Legnaro, Padova, IT

^c^ Department of Medicine and Surgery, Università di Perugia, P.le Gambuli, 06132 Perugia, Italy;

^d^ Department of Veterinary Medicine, Università di Perugia, Via San Costanzo 4, 06126 Perugia, Italy

^e^ TherioExpert LLc. and College of Veterinary Medicine, University of Nottingham, Nottingham, UK

^f^ Department of Physics and Geology, University of Perugia, Via Pascoli, 06123 Perugia, Italy

ᶢ Tyrus Veterinary Clinic, Via Aldo Bartocci, 1G, 05100 Terni, Italy

*Correspondence: [sara.pastore.medvet](mailto:angela.polisca@unipg.it)@gmail.com

**MIFlowCyt-EV Framework**

- 1. Semen was collected as previously described (Johnston, 1991), from German Shepherd dogs aged from 2 to 4.5 years. The ﬁrst, second and third fractions of the ejaculate were separated into three 15ml vials (Falcon Conical, Corning). Within 1 hour the third fraction was centrifuged at 300xg and the supernatant was transferred into a clear tube and immediately vertically frozen at -80°C. The centrifuged third fraction was thawed, diluted (1:1 v/v) with 30 mM Tris–HCl and 130 mM NaCl buﬀer pH 7.6 centrifuged at 5000g for 30 min at 4°C to eliminate cell debris and residual spermatozoa (whole third fraction). The pellet was discarded, and the supernatant was centrifuged at 100,000g at 4°C in an Optima TLX ultracentrifuge with a swinging-bucket 60Ti rotor (Beckman Coulter, Brea, CA, USA) for 2 h at 4°C. The pellet was resuspended in PBS and diluted to obtain a final concentration of 1.0 – 1.5 mg of protein per ml. The EVs preparation was stored at -80°C until use. In some experiment the whole third fraction was used for the experiments, with no ultracentrifugation step performed.

All animals were conﬁrmed healthy based on history and clinical examination which included blood analysis (complete blood count analyses and biochemical analyses), andrological evaluation, and ultrasound exam of the prostate and testis (MyLab 30 Gold system Esaote; Genoa, Italy equipped with a 5.5–7.5 MHz microconvex probe).

- 1. 1.1 Aim: To analyse the presence of CD13 and CD26 in EVs of the third fraction of the ejaculate of German Shepherd dogs. We hypothesize that canine EVs should express CD13 and CD26.

1.2 Keywords: EV; healthy German Shepherd dog, CD13, CD26. 1.3 Experimental variables: All animals were healthy and in a narrow age interval. Scatter-based triggering was used for the detection of particles.

2.1 The presence of CD13 and CD26 was determined using antibody staining. 300μl of isolated EVs or whole third fraction were incubated with 5 µL anti-human CD13 Monoclonal Antibody conjugated with Alexa Fluor 488CD (Molecular probes #MHCD1320) and/or with 5μl of anti-human CD26 Monoclonal Antibody (BA5b), PE conjugated (Molecular probes #cd2604) for one hour at 20°C and protected from light. At the end of the incubation samples were diluted 1:2 in PBS.

3.1 A buffer-only control of 0.1 µm-filtered PBS was recorded at the same flow cytometer acquisition settings as all other samples, including triggering threshold, voltages, and flow rate. The buffer-only control had a count of ~10 events s−1.

3.3 Unstained controls were measured at the same dilution as stained samples. Flow cytometer acquisition settings were maintained for all samples, including triggering threshold, voltages, and flow rate. No substantial changes in scatter or fluorescence signals were observed between unstained and stained samples.

3.5 Single-stained controls were included. Flow cytometer acquisition settings were maintained for all samples, including triggering threshold, voltages, and flow rate. No substantial changes in scatter or fluorescence signals were observed between unstained and stained samples.

4.1 The flow cytometer was equipped with solid laser with a 488-nm filter a 95% reduction filter, a 530/505nm band pass filter and a 575/550nm band pass filter to detect CD13 (FITC) and CD26(PE) stained EVs, respectively. Data were collected on log scale histograms at a rate of 5,000–10,000 events per second. Populations were gated using forward/side scatter (linear scale) to identify EVs and then by their fluorescent properties (log scale) in accordance with fluorophore’s properties.

4.4 Calibration of the flow cytometer was performed using CST beads consisting in equal quantities of 3-μm bright, 3-μm mid, and 2-μm dim polystyrene beads that are dyed with fluorochromes (BD bioscience). Each EVs was represented by a point in a rectangular co-ordinate system, based on its light-scattering properties (Mezzasoma et al., 2022). The position and dimension of the gate was placed in line with that of isolated EVs.

5.1 EVs dimension, number and concentration were not analysed.

6.1 The MIFlowCyt checklist has been completed and attached in Supplementary Information.
